# Contribution of information about acute and geriatric characteristics to decisions about life-sustaining treatment for old patients in intensive care

**DOI:** 10.1186/s12911-022-02094-z

**Published:** 2023-01-06

**Authors:** Michael Beil, P. Vernon van Heerden, Dylan W. de Lange, Wojciech Szczeklik, Susannah Leaver, Bertrand Guidet, Hans Flaatten, Christian Jung, Sigal Sviri, Leo Joskowicz

**Affiliations:** 1 Department of Medical Intensive Care, Hadassah Medical Centre and Faculty of Medicine, Hebrew University of Jerusalem, Jerusalem, Israel; 2 Department of Anaesthesia, Intensive Care and Pain Medicine, Hadassah Medical Centre and Faculty of Medicine, Hebrew University of Jerusalem, Jerusalem, Israel; 3 Department of Intensive Care Medicine, University Medical Centre, University Utrecht, Utrecht, The Netherlands; 4 Department of Intensive Care, Jagiellonian University Medical College, Kraków, Poland; 5 Intensive Care, St George’s University Hospitals NHS Foundation Trust, London, UK; 6 Service de Réanimation Médicale, Hôpital Saint-Antoine, Assistance Publique Hôpitaux de Paris, Paris, France; 7 Intensive Care, Department of Clinical Medicine, Haukeland Universitetssjukehus, Bergen, Norway; 8 Department of Cardiology, Pulmonology and Vascular Medicine, Faculty of Medicine, Heinrich-Heine-University Duesseldorf, Moorenstraße 5, 40225 Duesseldorf, Germany; 9 School of Computer Science and Engineering, The Hebrew University of Jerusalem, Jerusalem, Israel

**Keywords:** Decision-making, Information theory, Intensive care, Life-sustaining treatment, Uncertainty

## Abstract

**Background:**

Life-sustaining treatment (LST) in the intensive care unit (ICU) is withheld or withdrawn when there is no reasonable expectation of beneficial outcome. This is especially relevant in old patients where further functional decline might be detrimental for the self-perceived quality of life. However, there still is substantial uncertainty involved in decisions about LST. We used the framework of information theory to assess that uncertainty by measuring information processed during decision-making.

**Methods:**

Datasets from two multicentre studies (VIP1, VIP2) with a total of 7488 ICU patients aged 80 years or older were analysed concerning the contribution of information about the acute illness, age, gender, frailty and other geriatric characteristics to decisions about LST. The role of these characteristics in the decision-making process was quantified by the entropy of likelihood distributions and the Kullback–Leibler divergence with regard to withholding or withdrawing decisions.

**Results:**

Decisions to withhold or withdraw LST were made in 2186 and 1110 patients, respectively. Both in VIP1 and VIP2, information about the acute illness had the lowest entropy and largest Kullback–Leibler divergence with respect to decisions about withdrawing LST. Age, gender and geriatric characteristics contributed to that decision only to a smaller degree.

**Conclusions:**

Information about the severity of the acute illness and, thereby, short-term prognosis dominated decisions about LST in old ICU patients. The smaller contribution of geriatric features suggests persistent uncertainty about the importance of functional outcome. There still remains a gap to fully explain decision-making about LST and further research involving contextual information is required.

*Trial registration*: VIP1 study: NCT03134807 (1 May 2017), VIP2 study: NCT03370692 (12 December 2017).

**Supplementary Information:**

The online version contains supplementary material available at 10.1186/s12911-022-02094-z.

## Background

Life-sustaining treatment (LST) in critically ill patients is considered inappropriate and, thus, should be withheld or withdrawn, when there is no reasonable expectation of an outcome that will be beneficial to these patients [1]. This is especially relevant in old patients for whom both baseline status and outcome are generally poorer than in younger cohorts [2, 3] and further functional decline might be detrimental for the self-perceived quality of life. Although prognostication in the intensive care unit (ICU) is notoriously difficult [4], it becomes pivotal when contemplating decisions about LST [5].

In old ICU patients, pre-existing geriatric characteristics such as progressive loss of functional independence are known to be of greater prognostic value in the long term than the severity of the acute illness [6–8]. Moreover, frailty as a correlate of functional disability [9] was shown to be an independent prognosticator in this particular patient population [10]. Despite these findings, there still is no detailed understanding how these features are integrated into the decision-making about LST. In fact, the substantial variability of these decisions—even within the same ICU [11]—suggests considerable uncertainty or missing information in this field [12] with significant ethical and legal implications [13, 14]. Since the amount of uncertainty is inversely related to that of information [15], measuring information involved in decision-making can be an important step towards a solution for this problem.

**Fig. 1:**
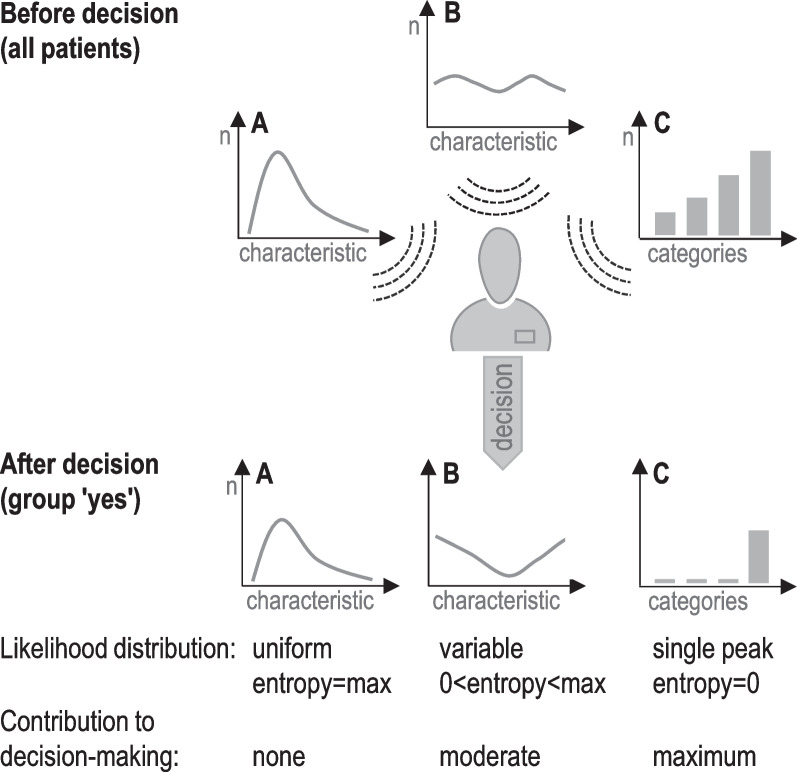
Analysis of information processing during decision-making. Methods from the framework of information theory are applied to quantify the differential contribution of patient characteristics to binary decision-making (yes/no). Shannon's entropy [15] of the likelihood distribution with regard to a specific decision is considered a measure of information used for that decision. Zero entropy indicates maximum information and minimum uncertainty. Note that the concept of entropy is related to that of variance for some types of distributions. In scenario **A**, the distribution of a continuous patient characteristic (e.g. age) does not change in response to the decision. Thus, the likelihood distribution is constant (uniform) and this characteristic is considered uninformative for that decision. Scenario **B** depicts a characteristic that partially contributes to decision-making. The extent of this contribution is measured by the entropy of the (non-uniform) likelihood distribution. In scenario **C**, the discrete patient characteristic is decisive, i.e. uncertainty is resolved by maximum information about categories

This study investigated the differential contribution of information about age, gender, frailty and other geriatric characteristics as well as severity of critical illness to decisions about LST in old patients admitted to ICU. We have analysed data from two multicentre studies (VIP1, VIP2) which recorded a variety of clinical characteristics, interventions and decisions about withholding or withdrawing LST in very elderly intensive care patients (VIP) [10, 16]. The current investigation aimed to measure the actual information contributed by these patient characteristics to get a detailed understanding of the decision-making process itself (Fig. 1). We applied techniques from the field of information theory [17] to meet the challenge of quantifying information during that process and extracting generalizable measures to compare variables of heterogeneous types [15].

## Methods

Datasets were obtained from two independent prospective observational studies (VIP1, VIP2) in ICU patients aged 80 years or older which documented decisions about withholding or withdrawing LST [10, 16]. The objectives of these multi-centre studies were to describe the prevalence of frailty (VIP1) and other geriatric conditions (VIP2) in that patient population on admission to ICU (baseline) and to assess their influence on short-term survival. The definitions used for the core baseline characteristics under investigation (age, gender, frailty, severity of the acute illness) were identical in both VIP1 and VIP2. Frailty was assessed by the clinical frailty scale (CFS) [18]. The sequential organ failure assessment (SOFA) score on admission to ICU was used to quantify the baseline severity of the acute illness [19]. Only patients with non-elective admissions and complete data with respect to these core characteristics were considered for further analysis. To investigate the impact of additional geriatric characteristics, we extracted a sub-group of patients from the VIP2 dataset having less than 20% missing data regarding the number of chronic co-morbidities, the patients' residence prior to hospital admission and the Katz index of independence in activities of daily living [10]. Note that these variables were only recorded in the VIP2 study.

Values for age, CFS (9 categories), SOFA score and the number of co-morbidities were binned into 8 categories each (2 years of age per bin, e.g. 80 and 81, with patients older than 95 years assigned to bin 8; 1 category of CFS per bin with CFS of 9 assigned to bin 8; 2 points of SOFA score per bin with SOFA scores greater than 15 assigned to bin 8; 1 count of co-morbidity per bin with counts greater than 7 assigned to bin 8). The Katz index ranges from 0 to 6 resulting in 7 bins for this variable. The patients' residence prior to hospital admission was classified into 4 categories: home, home with caregivers, nursing home or hospital, other.

Using the framework of information theory, the quantity of information within a probability distribution *P* of a discrete variable *X* with *N* mutually distinct states (categories) *x*_*i*_ can be described by Shannon's entropy *H(X)* [15]:

1 $$H\left( X \right) = - \sum\limits_{i} {p\left( {x_{i} } \right) \log_{2} p\left( {x_{i} } \right)\;{\text{with}} \;i = 1 \ldots N, \;0 \le p\left( {x_{i} } \right) \le 1} \;{\text{and}}\;\sum\limits_{i} {p\left( {x_{i} } \right) = 1}$$

Note that the base of the logarithm is usually set to 2 so that the unit of information is 1 bit. In this case, *H(X)* represents the minimum number of bits necessary to encode all information contained in *P*. The minimum entropy of *P* is 0 if *p(x*_*i*_*)* = 1 and *p*(*x*_*j ≠ i*_) = 0. Minimum entropy defines a state of maximum information about *X*. In contrast, maximum entropy is equal to log_2_(*N*) when *x*_*i*_ = *N*^−1^ for all *N* states of *X* (uniform distribution). This represents a state of minimum information. Shannon's entropy is a global characteristic of *P*. In contrast to other information measures, it does not take local properties, e.g. the neighbourhood of local extrema, into account. Thus, there is no requirement to consider a specific relationship between different states *x*_*i*_ of *X*, e.g. being equidistant.

Reduction of entropy *H(X)*, i.e. increase of information, can be linked to gain in predictability *Π* which is bounded above by [20]:

2 $$H\left( X \right) = \Pi \log_{2} \Pi - \left( {1 - \Pi } \right)\log_{2} \left( {1 - \Pi } \right) + \left( {1 - \Pi } \right)\log_{2} \left( {N - 1} \right)\;{\text{with}}\; 0 < \Pi < 1$$

This equation describes the upper limit of predictability and is not related to a particular algorithm. By mapping the entropy *H(X)* onto a standardised scale, this equation provides a measure that can be used to compare information contained in distributions of different types of variables.

The (dis)similarity between two distributions *P* and *Q* of the same variable *X* (e.g. patient characteristic before and after decision-making) was measured by the Kullback–Leibler divergence:

3 $$D_{KL} \left( {P||Q} \right) = \sum\limits_{i} {p\left( {x_{i} } \right) \log_{2} \left( {p\left( {x_{i} } \right) q\left( {x_{i} } \right)} \right)}$$

When the base of the logarithm is set to 2, the divergence of *P* from *Q* is measured in bits (see above). Identity of *P* and *Q* is expressed by *D*_*KL*_ = 0. Furthermore, divergence of *P* and *Q* can be characterised by the area under the receiver operating characteristic (AUROC). An AUROC value of 0.5 indicates a lack of divergence between *P* and *Q*.

Bootstrapping (n = 100) was applied to estimate the accuracy of *H(X)* and *D*_*KL*_ by the standard deviation of random samples from the study populations.

Logistic regression using all available patient characteristics was employed as an alternative method to determine the relative impact (odds ratios) of variables on outcome, i.e. decisions about LST.

All analyses were performed using the R software package (version 4.0.4, www.r-project.org).

## Results

This study involved a total of 7488 patients from two independent studies (VIP1, VIP2) who were acutely admitted to ICU. Size and characteristics of patient groups are listed in Table 1. First, we examined if the distribution of patient characteristics were similar in both studies. Although the distribution of age was not different between VIP1 and VIP2, the distribution of frailty and SOFA score differed significantly in the Kolmogorov–Smirnov test (*p* < 0.01).

**Table 1:** Patient characteristics (median and interquartile range within group)

	VIP1 study			VIP2 study		
	All patients (n = 3727)	Withholding decision (n = 1070)	Withdrawing decision (n = 578)	All patients (n = 3761)	Withholding decision (n = 1116)	Withdrawing decision (n = 532)
Age (years)	84 (81–87)	84 (82–87)	84 (82–87)	84 (81–87)	84 (82–88)	84 (81–86)
Gender (% female)	48.2%	49.2%	43.6%	46.7%	47.7%	44.4%
Frailty (CFS)	4 (3–6)	5 (4–6)	5 (3–6)	4 (3–6)	4 (3–6)	4 (3–6)
SOFA score	7 (4–11)	8 (5–11)	10 (7–13)	6 (4–9)	7 (4–10)	8 (6–11)
Other geriatric features:	Not available	Not available	Not available	(n = 3358)	(n = 961)	(n = 405)
Functional impairment (Katz index)				6 (4–6)	6 (4–6)	6 (5–6)
Co-morbidities (number)				4 (3–5)	4 (3–6)	4 (3–5)
Residence (number)						
Home				2510	683	301
Home with caregiver				357	109	37
Nursing home/hospital				451	157	66

**Fig. 2:**
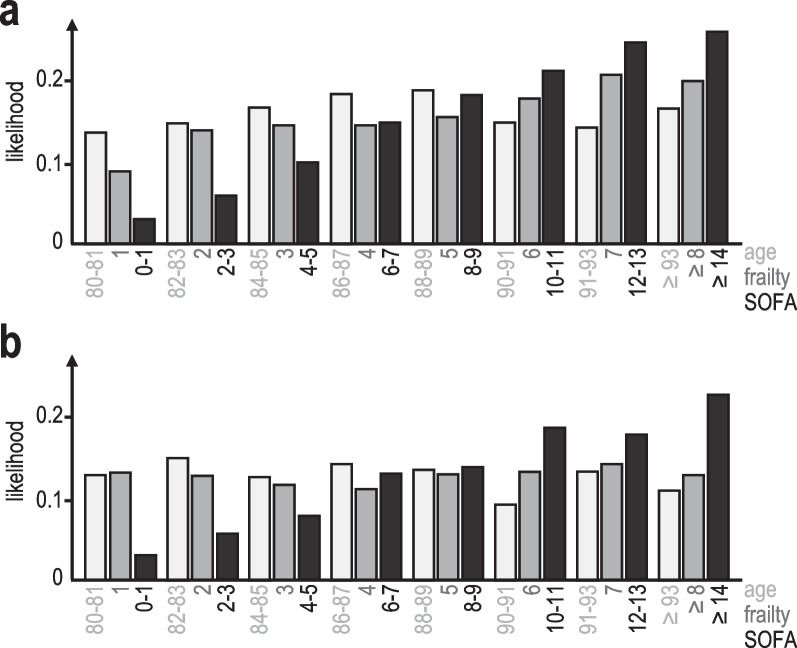
Likelihood of decisions to withdraw LST in the VIP1 (**a**) and VIP2 study (**b**). Likelihood ratios are shown for core patient characteristics: age (years) in light grey, frailty (CFS) in dark grey, SOFA score in black

The likelihood of decisions about LST was determined for each category of discrete patient characteristics (Fig. 2). The information contained in the likelihood distribution is measured as entropy *H(X)* (Table 2). Additional file 1: Fig. S1 illustrates the relationship between that distribution and *H(X)*. The lowest value for *H(X)*, i.e. the largest amount of information and smallest uncertainty of the likelihood distribution, was consistently found for the SOFA score in case LST was withdrawn (Table 2). Entropy values of 2.78 (VIP1) and 2.80 (VIP2) translate into upper bounds of predictability *Π* of 0.34 and 0.33, respectively. This means that at best 34% and 33% of patients can be accurately linked to specific SOFA scores in case of withdrawing decisions. Other patient characteristics showed a smaller influence on decision-making (Table 2). The entropy values of frailty correspond to a best possible predictability of 0.26 and 0.22 for withholding decisions in VIP1 and VIP2, respectively. Note that for maximum entropy *H*_*max*_*(X)* indicating a uniform likelihood distribution, predictability reaches a minimum, e.g. 0.125 for 8 categories. Entropy values close or equal to *H*_*max*_*(X)* suggest that a particular patient characteristic was considered minimally or uninformative during decision-making. Several of the scenarios listed in Table 2 approached that situation. We also examined the relative impact of patient characteristics on LST decisions by logistic regression based on all available variables. Although the results provided by information theory cannot be translated directly into odds ratios, there is a consistent trend with higher odds ratios being associated with larger decreases of *H(X)* for specific variables, i.e. amount of information used for decision-making (Table 2).

**Table 2:** Quantity of information of likelihood distributions for decisions about LST as measured by its entropy *H(X)* (mean ± standard deviation for multiple samples obtained by bootstrapping)

	*H*_*max*_	Withholding decision		Withdrawing decision	
		VIP1	VIP2	VIP1	VIP2
Age	3.0	2.98 ± 0.003 (1.04, 1.02–1.06)	2.97 ± 0.004 (1.06, 1.04–1.08)	2.99 ± 0.005 (1.03, 1.0–1.05)	2.98 ± 0.009 (0.99, 0.82–1.02)
Gender (female vs male)	1.0	1.0 ± 0.0004 (0.98, 0.84–1.13)	1.0 ± 0.0004 (1.02,0.88–1.18)	0.99 ± 0.002 (0.79, 0.66–0.95)	1.0 ± 0.001 (0.99, 0.82–1.2)
Frailty (CFS)	3.0	2.91 ± 0.009 (1.22, 1.17–1.27)	2.95 ± 0.006 (1.15, 1.1–1.20)	2.96 ± 0.01 (1.08, 1.03–1.14)	2.99 ± 0.003 (1.0, 0.95–1.05)
SOFA	3.0	2.97 ± 0.005 (1.03, 1.01–1.05)	2.94 ± 0.007 (1.07, 1.05–1.09)	2.78 ± 0.02 (1.12, 1.10–1.15)	2.80 ± 0.02 (1.14, 1.12–1.17)
Other geriatric features:		Not available		Not available	
Co-morbidities (number)	3.0		2.98 ± 0.003 (1.02, 0.99–1.06)		2.97 ± 0.01 (0.98, 0.93–1.02)
Functional impairment (Katz index)	2.8		2.78 ± 0.004 (1.01, 0.96–1.07)		2.79 ± 0.007(1.05, 0.97–1.14)
Residence (home - baseline)*	2.0		1.99 ± 0.004		1.97 ± 0.02
Home with caregiver			(0.8, 0.61–1.03)		(0.68, 0.46–0.99)
Nursing home/hospital			(1.05, 0.83–1.32)		(1.15, 0.84–1.56)

Note that the maximum entropy *H*_*max*_ varies according to the number of bins for each variable. Odds ratios (per 1-point increase) and 95% confidence intervals from logistic regression are depicted in brackets to show the influence of specific variables in these decisions

^*^Note that *H(X)* describes the distribution of probabilities for all residence categories, whereas the odds ratios are determined for comparisons between two specific types of residence

Although the number of patients per country was not large enough to obtain sufficiently robust results for systematic comparisons, two countries contributed more than 500 patients in one study and this pair was used as an example to assess effects by potentially variable preferences. *H(X)* was found to be similar between these two countries for all core characteristics with regard to withholding decisions (age: 2.95 ± 0.02 vs 2.95 ± 0.02, gender: 0.99 ± 0.007 vs 1.0 ± 0.001, CFS: 2.88 ± 0.03 vs 2.85 ± 0.03, SOFA: 2.88 ± 0.02 vs 2.88 ± 0.03), but differed substantially for frailty regarding withdrawing decisions (age: 2.91 ± 0.04 vs 2.87 ± 0.09, gender: 0.99 ± 0.004 vs 0.99 ± 0.007, CFS: 2.97 ± 0.02 vs 2.72 ± 0.02, SOFA: 2.70 ± 0.03 vs. 2.75 ± 0.06).

To validate the findings in Table 2, we determined the Kullback–Leibler divergence *D*_*KL*_ for the distributions of patient characteristics before and after decisions about LST (Table 3). Larger shifts indicate a greater contribution of a particular characteristic to the decision-making. For both VIP1 and VIP2, the largest shifts were found for the SOFA score with respect to withdrawing decisions. Very small values of *D*_*KL*_ for gender indicate the absence of biases for that patient characteristics in line with the data in Table 2. These findings were further corroborated by estimates from ROC curve statistics used to assess discrimination between distributions of patient characteristics after binary decisions (Table 3).

**Table 3:** Kullback–Leibler divergence *D*_*KL*_ between distributions of patient characteristics before and after decisions about LST (mean ± standard deviation for multiple samples obtained by bootstrapping)

	Withholding decision		Withdrawing decision	
	VIP1	VIP2	VIP1	VIP2
Age	0.02 ± 0.003 (0.55)	0.02 ± 0.003 (0.56)	0.01 ± 0.003 (0.53)	0.01 ± 0.004 (0.51)
Gender (female vs male)	0.0004 ± 0 (0.51)	0.0003 ± 0 (0.51)	0.006 ± 0.003 (0.53)	0.002 ± 0.001 (0.51)
Frailty	0.07 ± 0.006 (0.61)	0.04 ± 0.005 (0.59)	0.02 ± 0.005 (0.55)	0.007 ± 0.002 (0.51)
SOFA	0.02 ± 0.003 (0.55)	0.05 ± 0.005 (0.59)	0.2 ± 0.02 (0.66)	0.2 ± 0.02 (0.66)
Other geriatric features:	Not available		Not available	
Co-morbidities (number)		0.02 ± 0.002 (0.55)		0.01 ± 0.004 (0.51)
Functional impairment (Katz)		0.07 ± 0.007 (0.57)		0.02 ± 0.01 (0.51)
Residence		0.002 ± 0.001 (0.52)		0.01 ± 0.004 (0.50)

Discrimination between these distributions was also assessed by ROC curve analysis (AUROC in brackets)

**Fig. 3:**
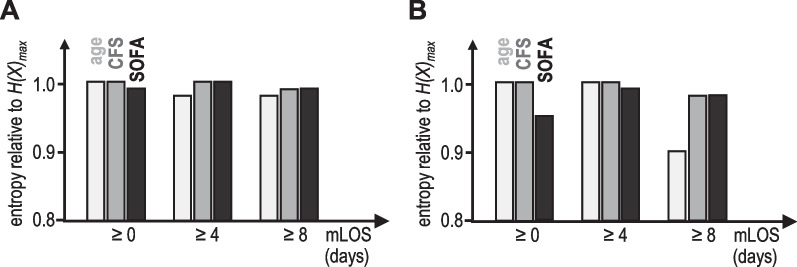
Decision-making about LST and length of stay in ICU. Relative contribution of age (light grey), frailty (CFS, dark grey) and SOFA score (black) to decisions of withholding (panel **A**) or withdrawing (panel **B**) LST for frail patients (CFS ≥ 4) and different minimal lengths of stay (mLOS) in ICU. Note that the smaller the entropy *H(X)* is, the larger is the contribution of a specific patient characteristic to decision-making

Finally, we investigated if the information processed for decision-making changes the longer vulnerable (frail) patients stay in ICU. Figure 3 depicts the entropy *H(X)* of the likelihood distributions for age, frailty and SOFA for these patients according to the type of decision and the length of stay. These data demonstrate that age becomes an important factor for the decision to withdraw LST after approximately 1 week in ICU. The SOFA score taken on admission predictably loses its importance for these decisions over time.

## Discussion

This study investigated the specific contribution of acute and geriatric patient characteristics to decisions about withholding or withdrawing LST in very old patients admitted to ICU with acute illnesses. Although some authors argue that there is no ethically relevant difference between withdrawing and withholding LST, there might be legal concerns about decisions to intentionally withdraw LST which may hasten death [21]. We analysed information with regard to the type of decision as an outcome in itself and did not examine the ethical or legal appropriateness or accuracy of these decisions.

Information about the severity of the acute illness on admission to ICU influenced decisions about withdrawing LST in old patients to a larger degree than age, gender and pre-existing frailty or other geriatric characteristics did. This can be interpreted as a lower degree of uncertainty felt in the decision-making with respect to the disease severity. That result was largely consistent in its extent for two independent datasets (VIP1, VIP2). Moreover, it confirms a pattern previously described in an observational study involving adult patients of all age groups [22]. These findings were, in principle, further validated by logistic regression, which demonstrated a correlation between the extent of odds ratios and the amount of information about specific characteristics used during decision-making. In contrast to logistic regression, however, information theory can also provide valid insights for non-monotonic relationships between variables and outcome (decisions). Moreover, that approach summarises the contribution of information by multi-categorical variables, such as type of residence, in a single number.

Small differences of entropy values for likelihood distributions to maximum entropy might suggest small effect sizes. However, the underlying deviations from the uniform and uninformative distribution can be substantial and indicate some degree of informed decisions about LST (Additional file 1: Fig. S1). Of note, a fully informed decision, in which any uncertainty about the specific role of a patient characteristic is (unrealistically) removed, would result in a single peak of the distribution and, thus, entropy would approach zero.

Although our findings show a maximum of uncertainty for some characteristics, such as gender, others were found to be informative for decision-making to a variable extent which can be ranked by entropy. The large effect size for the SOFA score emphasises its prominent role in withdrawing decisions. This result was surprising, especially regarding previous studies about the substantial impact of pre-existing (geriatric) disabilities on outcome in these patients [6, 7]. Of note, there was a difference between two countries with respect to the specific role of frailty. This pre-liminary finding suggests some variability of opinions about the prognostic importance of frailty, which should be further investigated in future studies. In general though, triage prior to ICU admission might have selected a particular population of old patients for intensive care where frailty and other geriatric conditions were considered less important for prognostication than other, acute and seemingly reversible problems. A similar selection process could have happened in regard to age that apparently turned this characteristic into an almost uninformative parameter for decisions about LST in old patients. However, a sub-group analysis revealed that age becomes an important factor for withdrawing decisions in the specific cohort of frail patients being more than a week in ICU. Of note, the more pronounced findings with respect to withdrawing decisions are not unexpected since this particular decision is considered more distinctive and legally demanding than withholding decisions which are defined and implemented in variable ways [11].

Although information about the SOFA score played a prominent role in the decision-making, it does not fully explain withdrawing decisions. This is not surprising and underlines that other variables such as contextual and organisational factors influenced decision-making. These include personal preferences of medical professionals as well as those of patients or surrogate decision-makers [23]. Preferences evolve during ICU admission when both the response to interventions and their burden for the individual patient become visible. These changes may also explain the growing importance of age for withdrawing decisions in frail patients after a week in ICU.

The above findings illustrate how methods from information theory help to identify specific patient groups, such as very old individuals, which had been managed differently by implementing preference-based or biased decisions about LST. These techniques can also help to design audits and monitor changes of clinical behaviour. Furthermore, by measuring the amount of information contributed to decision-making by known variables, i.e. effect size, we can also estimate the relative impact of additional factors on specific decisions. Trade-offs between conflicting interests concerning a particular outcome as expressed by different variables can be characterised by measuring the relative impact of these variables [12]. For example, our results suggest that most intensivists participating in VIP1 and VIP2 were more focused on short-term outcome than functional capacity in the long term which might have been considered more uncertain.

The implications of this study for clinical practice are:

(i) Frailty constituted a new concept in ICU at the time of VIP1 and VIP2 and showed a less than expected role in decision-making about LST. This implies that its use in clinical practice may benefit from further research and educational efforts.
(ii) More contextual information about patients might be required to further reduce uncertainty in decisions about LST. Although this comes at a cost, the benefit of uncertainty reduction from an ethical as well as legal point of view could provide sufficient justification for additional expenses as emphasised by events during the COVID-19 pandemic [14, 24]. In general, quantification of information and uncertainty improves the transparency of decision-making processes and supports standardisation of decisions [25, 26].
(iii) The contribution of chronological age to decision-making within the examined cohorts in ICU was very small, except for frail patients being in ICU for more than a week. In general, monitoring for potentially inappropriate preferences (biases) can help to ensure non-discriminatory access to healthcare resources [27, 28].

Major limitations of this study were:

(i) The VIP1 and VIP2 studies were not primarily designed to analyse decision-making about LST. The set of variables recorded in these studies did not provide highly granular contextual features known to contribute to that process, notably personal preferences of physicians, patients and surrogate decision-makers which may reflect social and geographic variations [29].
(ii) The knowledge about frailty and other geriatric characteristics and their impact on the prognosis of critical conditions is evolving. So is its transfer into clinical practice [24]. Thus, a larger contribution of frailty and probably other functional characteristics to decision-making can be expected in due time.
(iii) This study focused on the role of individual patient characteristics in the decision-making. Future studies may examine the impact of combinations of clinical variables on decisions about LST.

## Conclusions

For old ICU patients recruited to the VIP1 and VIP2 study, the severity of the acute illness contributed to decision-making about LST withdrawal to a larger extent than age, frailty or other geriatric characteristics. In this particular patient group, however, frailty is known to substantially influence functional outcome in the long term and, thus, should be considered for LST decisions to a greater degree. Further research involving more contextual information will have to elucidate underlying reasons.

To the best of our knowledge, this is the first investigation of decisions about LST in ICU based on methods from the field of information theory. This framework provides techniques to quantitatively assess decision-making [17] and complements methods based on classical statistics, such as logistic regression [30]. However, the absence of major constraints concerning data distributions as well as the availability of a standardised scale for information from different types of variables provides a considerable benefit for comparative data analysis. In the future, this might especially benefit 'big data' techniques and high-dimensional modelling approaches from artificial intelligence [31].

## Supplementary Information

**Additional file 1**: **Fig. S1**. Hypothetical likelihood distributions for LST decisions with regard to a patient characteristic with 8 distinct categories. The three examples depict simulated distributions with decreasing values for the entropy *H*(*X*) which suggest an increasing contribution of that patient characteristic to these decisions. The more extensive preferences for selected categories are during decision-making, the smaller is *H*(*X*).

## Data Availability

The datasets analysed during the current study are not publicly available due to contractual restrictions but are available from the corresponding author on reasonable request.

## Abbreviations

AUROC: Area under the receiver operating characteristic
CFS: Clinical frailty scale
ICU: Intensive care unit
LST: Life-sustaining treatment
SOFA: Sepsis-related organ failure assessment
