# Supplementary material for: Contribution of information about acute and geriatric characteristics to decisions about life-sustaining treatment for old patients in intensive care
Source: BMC Med Inform Decis Mak. 2023 Jan 6;23:1. doi: 10.1186/s12911-022-02094-z (PMC9818057; doi:10.1186/s12911-022-02094-z)

Supplementary material

Figure S1.
Hypothetical likelihood distributions for LST decisions with regard to a patient characteristic with 8 distinct categories. The three examples depict simulated distributions with decreasing values for the entropy *H(X)* which suggest an increasing contribution of that patient characteristic to these decisions. The more extensive preferences for selected categories are during decision-making, the smaller is *H(X)*.

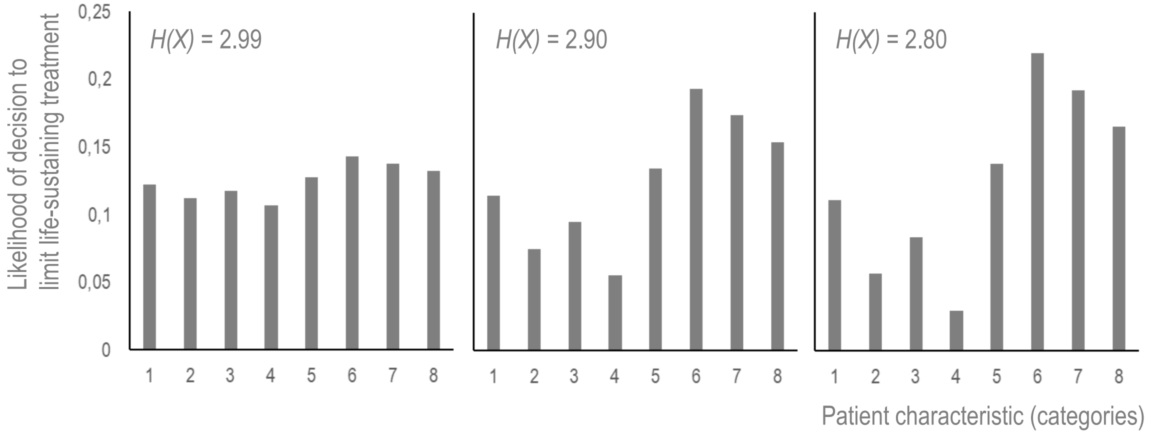

Supplement: Supplementary file 1 — Additional file 1: Fig. S1. Hypothetical likelihood distributions for LST decisions with regard to a patient characteristic with 8 distinct categories. The three examples depict simulated distributions with decreasing values for the entropy H(X) which suggest an increasing contribution of that patient characteristic to these decisions. The more extensive preferences for selected categories are during decision-making, the smaller is H(X). [file 12911_2022_2094_MOESM1_ESM.pdf]
